# Trends in horizontal inequity in access to public health care services by immigrant condition in Spain (2006–2017)

**DOI:** 10.1186/s12939-019-1092-1

**Published:** 2019-11-29

**Authors:** Jaime Pinilla, Miguel A. Negrín, Ignacio Abásolo

**Affiliations:** 1 Departamento de Métodos Cuantitativos en Economía y Gestión, Universidad de Las Palmas de Gran Canaria, Las Palmas, Spain; 2 Departamento de Economía Aplicada y Métodos Cuantitativos, Instituto Universitario de Desarrollo Regional, Universidad de La Laguna, La Laguna, Santa Cruz de Tenerife Spain; 3 Facultad de Economía, Empresa y Turismo, Campus de Guajara, 38071 La Laguna, Santa Cruz de Tenerife Spain

**Keywords:** Horizontal equity in access, Economic immigration, Public health care services, National health surveys

## Abstract

**Background:**

The objective of this research is to analyse trends in horizontal inequity in access to public health services by immigration condition in Spain throughout the period 2006–2017. We focus on “economic immigrants” because they are potentially the most vulnerable group amongst immigrants.

**Methods:**

Based on the National Health Surveys of 2006–07 (*N* = 29,478), 2011–12 (*N* = 20,884) and 2016–17 (*N* = 22,903), hierarchical logistic regressions with random effects in Spain’s autonomous communities are estimated to explain the probability of using publicly-financed health care services by immigrant condition, controlling by health care need and other socioeconomic and demographic variables.

**Results:**

Our results indicate that there are several horizontal inequities, though they changed throughout the decade studied. Regarding primary care services, the period starts (2006–07) with no global evidence of horizontal inequity in access (although the analysis by continent shows inequity that is detrimental to Eastern Europeans and Asians), giving way to inequity favouring economic immigrants (particularly Latin Americans and Africans) in 2011–12 and 2016–17. An opposite trend happens with specialist care, as the period starts (2006–07) with evidence of inequity that is detrimental to economic immigrants (particularly those from North of Africa) but this inequity disappears with the economic crisis and after it (with the only exception of Eastern Europeans in 2011–12, whose probability to visit a specialist is lower than for natives). Regarding emergency care, our evidence indicates horizontal inequity in access that favours economic immigrants (particularly Latin Americans and North Africans) that remains throughout the period. In general, there is no inequity in hospitalisations, with the exception of 2011–12, where inequity in favour of economic immigrants (particularly those from Latin America) takes place.

**Conclusions:**

The results obtained here may serve, firstly, to prevent alarm about negative discrimination of economic immigrants in their access to public health services, even after the implementation of the Royal Decree RD Law 16/2012. Conversely, our results suggest that the horizontal inequity in access to specialist care that was found to be detrimental to economic immigrants in 2006–07, disappeared in global terms in 2011–12 and also by continent of origin in 2016–17.

## Introduction

Spain has a National Health System (SNHS) characterised by universal coverage and tax funding, thus patients face a zero price at the point of consumption for most health care services although in order to access specialist or hospital care, patients must first visit a general practitioner (GP), who acts as a “gate-keeper” for the health system. The SNHS is decentralised into seventeen regional health care systems corresponding to the seventeen “autonomous communities” (hereinafter regions). The regions are very different in size and in population, ranging from 315,000 inhabitants in La Rioja to almost 8.5 million inhabitants in Andalusia. All of them have full authority over planning and regulating -always in compliance with the laws of the Spanish State - including the management of health care provision to their respective citizens (natives and immigrants).

One of the principles of the SNHS is that access to and provision of health care should be established in conditions of effective equality. According to this principle, a consistent horizontal equity criterion would be to ensure “equal access for equal health care need” (see for example [1]). Thus, other individual characteristics not related to health care need should not matter (eg. region of residence, income, education, immigrant condition, etc.). The analysis of horizontal equity in access to health care services by immigrant condition in Spain at the State level has been a matter of social concern as shown by previous evidence addressing this issue [2–10] or showing its relative importance together with other inequities in the access to provision of health care [11] . The period 2006–2017 is marked by two important events that might have affected access to public health care services in the SNHS, particularly for vulnerable population groups as is the case of economic immigrants. Namely, the economic crisis that started in 2008 and, second, a remarkable regulation change that took place in 2012 as a consequence of the crisis.

The availability of three waves of the Spanish National Health Survey for 2006–07, 2011–12 and 2016–17 allows us to carry out an empirical analysis of the evolution of equity in access to the SNHS by immigrant condition before, during and after both noteworthy events. First, regarding the economic crisis that started in 2008, although the main budget cuts affecting the SNHS did not start to take place until 2010 (and they were heterogeneously implemented by the seventeen regional health authorities), the 2011–12 survey may already give us evidence of the initial effects of the economic crisis on access to the SNHS by immigrant condition, particularly compared with the situation in 2006–07. Second, regarding the regulation of immigrant access to health care, the SNHS was characterised by universal access, handling all residents under the same conditions (included irregular immigrants registered in the municipal register, Organic Law OL4/2000). However, this changed in July 2012, when the Royal Decree RD Law 16/2012 established a different way of handling irregular immigrants over 18, who were just given access to emergency services and maternity services (only those aged below 18 could have comprehensive health care). However, the enforcement of this policy was not homogeneous throughout the Spanish territory and different regions implemented it to different degrees in the subsequent years. The 2016–17 survey may show evidence of the extent to which this regulation change is associated with a change in equity in access to the SNHS as compared with 2006–07 and 2011–12.

Access to the different health care services of the SNHS (i.e. primary care, specialist care, hospitalisations and emergency services), has different connotations. Since general practitioner (GP) and emergency services are patient-initiated demand services, access to them strongly depends on patients’ characteristics, preferences, perceptions of their health care need and expectations from the health system. Conversely, specialist care and hospitalisations are doctor-initiated demands, thus access to these types of services heavily depend on health care need as evaluated by the doctor, and ultimately is a matter for the SNHS organisation and rules.

Regarding previous evidence about access to health care services for Spain, Hernández-Quevedo and Jiménez-Rubio [3], using the national health surveys of 2003 and 2006–07 and estimating the probability of using each of the health care services through logit regression models (adjusting for health needs, socioeconomic level and other demographic characteristics that are known to affect health care utilisation), concluded that immigrants -as compared with Spaniards- are more likely to be treated in hospitals and emergency services but less likely to contact a specialist doctor or a GP. Antón and Muñoz de Bustillo [6], also using the national health survey of 2006–07, estimated the utilisation frequency through negative binomial and hurdle models, finding no statistically significant differences in the patterns of visits to GPs and hospital stays between natives and immigrants, and a lower (higher) access to specialists (emergency rooms) for immigrants with respect to Spaniards. Sanz et al. [8] used the data of the 2006–07 national health survey and logistic regressions to analyse frequency of use of health services by gender and immigration condition once adjusted for health need and different socioeconomic and demographic characteristics, concluding that, in general, immigrants use health services less frequently than natives, but there are some exceptions depending on gender and continent of origin: immigrant men (women) use health care services less frequently (similarly) than their Spanish counterparts, with Sub-Saharans being those who use services more frequently. García-Subirats et al. [9] used the 2006–07 and 2011–12 national health surveys and estimated Poisson regression models to compare the utilisation of the different health services between immigrant and native-born populations in Spain. In 2011, as compared with 2006, they found a relatively greater utilisation of GP services by immigrants and a considerable reduction in the utilisation gap between both populations for specialist care. Note that the above literature has addressed access to health care services considering overall services, that is, public and privately funded health care services.

Against this background, this research aims to analyse the trends of horizontal inequity in access to health care services in Spain in the period 2006–2017 by immigration condition taking into account the following points. First, this research is concerned with access to publicly-funded health care services as we regard this as most relevant when we address horizontal equity in health care utilisation. Second, given the relevance of regions in the SNHS, as they are the responsible for health care management and therefore ultimately responsible for patients’ access to health care in their territory, variability of access across them will be included in the analysis. Third, given the particular vulnerability of individuals who migrate to Spain for economic reasons, we will differentiate economic and non-economic immigrants, focusing our study just on the former. In addition, in a second analysis the continent of origin will also be considered to highlight any differences in access amongst economic immigrants. Fourth, data from the three waves of the Spanish National Surveys will be pooled in order to increase the number of observations regarding the immigrant collective and a year dummy will account for changes in the period. Finally, to the best of our knowledge, this is the first study that analyses the trend of equity in access to the SNHS in a period that stretches from 2006 to 2017, thus also addressing the potentially related consequences of the introduction of the RD Law 16/2012 in Spain.

## Data, variables and methods

### Data and variables

The database used in this research was built by pooling data from the Spanish National Health Survey (a representative survey of the Spanish population) for three waves: 2006–07 (*N* = 29,478), 2011–12 (*N* = 20,884) and 2016–17 (*N* = 22,903). They are face-to-face, cross-sectional population-based surveys that employ a three-stage, stratified-random design to identify samples of adults aged 16 or over (2011–12 and 2016–17 waves also include aged 15 but they were dropped for comparative purposes). The first-stage units are the census sections, that are stratified according to the size of the municipality to which the section belongs. The second-stage units are the main family households. The third stage units are chosen from a list of persons within the household who can be interviewed and asked to fill in the questionnaire at the time the survey is carried out. The sample structure allows therefore that irregular immigrants are also included in the survey (for more details, see [12]). Data on health care utilisation, the condition of immigrant, self-reported morbidity and other demographic and socioeconomic characteristics were collected.

Access to health care (our dependent variable) is proxied by means of whether or not the individual has used the publicly- funded health service in question for a given period (so we have four different models, one for each health service). Regarding GP and specialist care, our dependent variable has been built upon the information available for the last visit in the past four weeks. Particularly, respondents are asked about the nature of the visit (GP or specialist). In addition, they are asked whether the doctor was in the public health system, was from a private insurance company or was in a private consulting room; we considered the former case as a publicly-funded visit, whilst the second and third cases were considered as privately-funded visits.

Likewise, with respect to emergency visits and hospitalisations, respondents are asked whether they had used each service in the past twelve months. With respect to the last emergency visit, we considered it as publicly funded if the individual responded that it took place in a public hospital or in a public health care centre; and we considered it as privately funded if the individual responded that it took place in a private clinic or a private centre. Regarding the last admission to hospital we considered it as publicly funded if the individual responded that it was funded by the social security or by other civil servants’ mutuality (i.e. MUFACE, MUGEJU, ISFAS); and we considered it as privately funded if the individual responded that it was funded by private insurance or it was out of pocket funded. The surveys include information on whether the admission was caused by a labour (or caesarean). Given the different rate of labours amongst immigrants we have excluded them from hospitalisations.

With respect to the explanatory variables, our main variable relates to the condition of immigrant. Regarding the concept of immigrant population, the Spanish National Health Survey provides with information about the country of birth and also about nationality of respondents. We have taken the definition of the World Health Organisation that considers migrants as persons “… .who have left their country of birth to reside elsewhere” [13]. We take account of two versions of this variable. Regarding the first version we follow Carrasco-Garrido et al. [2] who distinguish between “economic immigrants” and “non-economic immigrants”. Particularly, we define “economic immigrants” as those who were born in Eastern Europe, Latin America, Africa or Asia. And “non-economic immigrants” would be those who were born in any other place with the exception of Spain (this latter group is considered a “control” as the focus of our study relates to economic immigrants). So, according to the first version of this explanatory variable we classify the sample population in three categories native Spaniards, economic-immigrants and non-economic immigrants. A second version aimed to be more detailed, classifying the sample as follows: in addition to Spaniards and non-economic immigrants, economic immigrants are disaggregated by continent of origin in five categories (Eastern Europe, Asia, Latin America, North Africa and the rest of Africa).

In line with previous related literature we assume that access to health care services is mainly determined by three groups of characteristics: medical need, socioeconomic status and demographic characteristics [2–10]. Medical need is proxied by different variables. First, self-reported measures of individuals’ health state: these include a categorical indicator that records whether individuals considered their general health during the twelve months prior to the survey to be ‘very good’, ‘good’, ‘fair’ and ‘poor’ or ‘very poor’. Second, a set of dummy variables indicating whether the respondents report the presence of any of the seven listed chronic conditions (hypertension, strokes, heart problems, diabetes, cholesterol, cancer or mental health). Additionally, a continuous variable indicating the number of chronic conditions not listed above was specified. Third, two dummy variables were used representing whether any acute illness restricts the normal activity of respondents or had confined them to bed in the previous two weeks, or whether they had had any accident in which they had been injured in the previous twelve months. Fourth, to measure mental health another continuous variable (GHQ-12) was used with the 12-item version of the General Health Questionnaire [14], indicating the mental health of the respondent in a scale from 0 (best possible state) to 12 (worst possible state) [15].

Socioeconomic status is proxied through three variables: education, social class and employment situation. Education is measured by a categorical variable indicating the highest level of schooling achieved by the respondent: no studies, primary studies, secondary studies and university studies. Social class of the reference person in the household is grouped into four categories based on the National Classification of Occupations: high social class (directors and managers with university degrees), medium-high social class (intermediate professions and self-employed), medium-low social class (skilled and partly-skilled occupations), and low social class (unskilled workers). Employment situation of the respondent is measured by a categorical variable with three possible activity statuses: employed (i.e. the individual is currently employed), unemployed (i.e. the individual is currently unemployed), retired (i.e. whether the individual is retired) or other situation (the individual is a student, homemaker or other inactive situation).

Regarding other controls, we have considered gender, age (in seven age groups), size of the municipality of residence (a dummy variable distinguishing those who are resident in municipalities of less than 50,000 inhabitants) and living with a partner.

Table 1 presents the variables considered in the analysis and their main descriptive statistics.

**Table 1:** Descriptive stats for each year and for the pooled sample

Type var.	Variable	Mean (SD) 2006 (*N* = 29,478)	Mean (SD) 2011 (*N* = 20,884)	Mean (SD) 2016 (*N* = 22,903)	Mean (SD) 2006–2016 (*N* = 73,265)	*p*-value*
Dep. Var	Primary_care	0.3203	0.2916	0.2846	0.3009	< 0.001
	Specialist_care	0.1307	0.1312	0.1101	0.1244	< 0.001
	Emergencies	0.2592	0.2408	0.2658	0.2560	< 0.001
	Hospitalisation	0.0912	0.0816	0.0801	0.0850	< 0.001
Demographic variables	Female	0.6050	0.5413	0.5416	0.5670	< 0.001
	Age 16–25	0.0838	0.0826	0.0703	0.0792	< 0.001
	Age 26–35	0.1586	0.1422	0.1057	0.1374	
	Age 36–45	0.2010	0.1884	0.1838	0.1920	
	Age 46–55	0.1624	0.1683	0.1812	0.1700	
	Age 56–65	0.1405	0.1501	0.1669	0.1515	
	Age 66–75	0.1352	0.1298	0.1444	0.1365	
	Age76–85	0.0983	0.1069	0.1092	0.1042	
	Age more than 85	0.0202	0.0318	0.0384	0.0292	
	Living in pair	0.6149	0.5896	0.5446	0.5857	< 0.001
Health state variables	Health bad-very bad	0.1047	0.0943	0.0970	0.0993	< 0.001
	Health fair	0.2745	0.2281	0.2411	0.2508	
	Health good	0.4801	0.5012	0.4828	0.4870	
	Health very good	0.1407	0.1764	0.1791	0.1629	
	Hypertension	0.2493	0.2581	0.2726	0.2591	< 0.001
	Stroke	0.0248	0.0226	0.0231	0.0237	0.230
	Heart	0.0711	0.0740	0.0849	0.0763	< 0.001
	Diabetes	0.0731	0.0889	0.0989	0.0857	< 0.001
	Cholesterol	0.1843	0.2177	0.2385	0.2108	< 0.001
	Tumor	0.0324	0.0353	0.0500	0.0388	< 0.001
	Mental	0.1685	0.1360	0.1547	0.1549	< 0.001
	Rest chronics	2.0445 (2.3064)	1.7700 (2.1561)	2.1319 (2.5000)	1.9936 (2.3324)	< 0.001
	Limitation 2 weeks	0.1574	0.1222	0.1486	0.1446	< 0.001
	Accidents 12 months	0.1030	0.0382	0.0421	0.0655	< 0.001
	GHQ12	1.6194 (2.6429)	1.5935 (2.7639)	1.4490 (2.7358)	1.5579 (2.7087)	< 0.001
Continent of birth	Native Spanish	0.9170	0.9140	0.9027	0.9116	< 0.001
	Economic immig.	0.0654	0.0707	0.0820	0.0721	
	Eastern Europe	0.0166	0.0145	0.0164	0.0139	
	Asian	0.0022	0.0034	0.0046	0.0033	
	Latin American	0.0372	0.0385	0.0414	0.0389	
	North Africa	0.0120	0.0112	0.0153	0.0128	
	Rest Africa	0.0022	0.0032	0.0042	0.0031	
	Non-econ immig.	0.0177	0.0153	0.0154	0.0163	
Socioeconomic variables	No studies	0.1393	0.1469	0.1196	0.1353	< 0.001
	Primary studies	0.3472	0.1269	0.1924	0.2358	
	Secondary studies	0.3618	0.5760	0.5050	0.4678	
	University studies	0.1517	0.1503	0.1830	0.1611	
	Low social class	0.1387	0.1504	0.1441	0.1437	< 0.001
	Medium-low SC	0.4140	0.4792	0.4859	0.4549	
	Medium-high SC	0.2572	0.1868	0.1905	0.2164	
	High social class	0.1901	0.1836	0.1796	0.1850	
	Employed	0.4480	0.4184	0.4331	0.4349	< 0.001
	Unemployed	0.0624	0.1259	0.1086	0.0950	
	Retired	0.2750	0.2547	0.2885	0.2734	
	Inactive	0.2146	0.2010	0.1698	0.1967	
Other	Small municipality	0.5566	0.4955	0.4992	0.5212	< 0.001

* Chi-square test for categorical variables and Kruskal-Wallis test for continuous variables

### Methods

We use a hierarchical (multilevel) logistic regression to estimate the probability of using each of the four health services (general practitioner, specialist, hospitalisation and emergencies). As it was mentioned above, the seventeen Spanish regions have the responsibility of the management of public health care, including the issues related to access to health care of their respective residents, thus a multilevel model seems more appropriate. The model for each health service can be written as follows:

$$ {y}_{ic}\sim Ber\left({p}_{ic}\right) $$

$$ \mathrm{Logit}\ \left[{p}_{ic}\right]={x}_i^{\prime}\beta +{I}_i^{\prime}\gamma +{\varepsilon}_c $$

$$ {\varepsilon}_c\sim N\left(0,{\sigma}_c^2\ \right) $$

Where y_ic_ is the endogenous variable for an individual _i_ who lives in region *c* and takes value 1 if the individual reports having used the health service, 0 otherwise. *xi* is the vector of explanatory variables (including an intercept) of the model for individual i. β is the vector of coefficients. *Ii* is the vector of explanatory variables related to immigration, which includes the interactions between the immigration indicator variables and every dummy variable representing the year, where the interaction between the Spaniard indicator variable and the year 2006–07 has been omitted, acting as a reference. ε_c_ is the random perturbance term corresponding to each region.

In order to test the null hypothesis that there is no inequity in access, we check the sign and statistical significance of γ (and combination of γ) that will indicate, for each of the health care services considered and of the three years, whether the probability of using the health service by the population group of economic immigrants is the same, greater or smaller than that for native Spaniards. Although non-economic immigrants are also included, we consider them merely as a control variable.

## Results

Table 1 shows the descriptive statistics for the variables of the study. For the pooled sample, the percentages for economic immigrants, native Spaniards and non-economic immigrant populations are 7.21, 91.16 and 1.63%, respectively (while the native population reduced its weight over the period analysed, the immigrant population increased slightly, particularly between 2011 and 12 and 2016–17, driven by North Africans and Latin Americans). We have used official data from the Spanish National Institute of Statistics [16] to calculate the corresponding real percentages using an average of the years 2006, 2011 and 2016, resulting in 9.86, 87.26 and 2.88%, respectively. Thus, as expected, economic immigrants are slightly under-represented in the Spanish national health surveys (as well as no-economic immigrants). In addition, as pointed by Carrasco-Garrido et al. [2], over representation of the Latin American population is likely to occur given that it is easier for them to answer the Spanish-written questionnaires of the survey. When we compare the percentage of the surveys with those of the official statistics, for Latin Americans, these figures are 3.89 and 4.74%, respectively (that is, only a 18% lower than the official statistics); for Eastern Europeans these figures are 1.39 and 2.19% (that is, a 36% lower); for Africans these figures are 1.59 and 2.19% (that is, a 28% lower); and for Asians the corresponding figures are 0.33 and 0.74% (that is, a 56% lower). Thus, the over representation of Latin Americans is confirmed for this three-year period analysis. Regarding the sample frequencies for the different health care services, it can be seen that over the period 2006–2017 there is a decrease in these figures for the four health care services, indicating a reduction in utilisation of the SNHS over the period analysed, with the only exception of emergency services that increases in 2016–17 reaching a higher value than the one of 2006–07.

Table 2 shows the sample frequencies for the independent variables for the pooled sample by each of the population groups (economic immigrants, natives and non-economic immigrants). There are no gender differences between the population groups. However, economic immigrants are a younger population as compared to natives (and also compared to non-economic immigrants). In addition, for all the other health indicators, economic immigrants report having a better state of health than Spaniards. Regarding socioeconomic status, the profile of the economic immigrant (as compared with natives) responds to someone with a relatively high education level but low social class. Unemployment is more frequent among economic immigrants (17.87%) than natives (8.83%).

**Table 2:** Descriptive stats by type of immigration for the pooled sample

Type var.	Variable	Economic Immigrants Mean (SD)	Native Spanish Mean (SD)	Non-Econ Immigrants Mean (SD)	*p*-value*
Demographic variables	Female	0.5675	0.5671	0.5591	0.956
	Age 16–25	0.1416	0.0743	0.0796	< 0.001
	Age 26–35	0.2950	0.1245	0.1609	
	Age 36–45	0.2880	0.1834	0.2506	
	Age 46–55	0.1630	0.1704	0.1769	
	Age 56–65	0.0712	0.1578	0.1526	
	Age 66–75	0.0240	0.1459	0.1123	
	Age76–85	0.0144	0.1122	0.0545	
	Age more than 85	0.0027	0.0316	0.0126	
	Living in pair	0.5607	0.5873	0.6086	< 0.001
Health state variables	Health bad-very bad	0.0519	0.1038	0.0595	< 0.001
	Health fair	0.2145	0.2548	0.1869	
	Health good	0.5007	0.4856	0.5013	
	Health very good	0.2329	0.1558	0.2523	
	Hypertension	0.1195	0.2715	0.1820	< 0.001
	Stroke	0.0081	0.0250	0.0168	< 0.001
	Heart	0.0282	0.0804	0.0553	< 0.001
	Diabetes	0.0360	0.0905	0.0386	< 0.001
	Cholesterol	0.1034	0.2202	0.1584	< 0.001
	Tumor	0.0150	0.0408	0.0302	< 0.001
	Mental	0.0854	0.1614	0.1014	< 0.001
	Rest chronics	1.1528 (1.7448)	2.0701 (2.3641)	1.4317 (1.9875)	< 0.001
	Limitation 2 weeks	0.1348	0.1458	0.1199	0.029
	Accidents 12 months	0.0506	0.0670	0.0478	< 0.001
	GHQ12	1.5107 (2.4806)	1.5676 (2.7310)	1.2142 (2.3649)	0.027
Socioeconomic variables	No studies	0.0815	0.1413	0.0331	< 0.001
	Primary studies	0.1571	0.2441	0.1196	
	Secondary studies	0.6103	0.4551	0.5496	
	University studies	0.1511	0.1595	0.2977	
	Low social class	0.2940	0.1329	0.0843	< 0.001
	Medium-low SC	0.5052	0.4525	0.3635	
	Medium-high SC	0.1054	0.2250	0.2274	
	High social class	0.0955	0.1896	0.3248	
	Employed	0.5927	0.4213	0.4954	< 0.001
	Unemployed	0.1787	0.0883	0.0947	
	Retired	0.0441	0.2920	0.2506	
	Inactive	0.1850	0.1992	0.1601	
Other	Small municipality	0.4344	0.5273	0.5658	< 0.001

* Comparison Economic Immigrants vs. Native Spanish. Chi-square test for categorical variables and U-Mann Whitney test for continuous variables

Table 3 presents the sample frequencies by the dependent variables over the three years and also for the pooled sample. For the pooled sample, whilst the frequencies for GPs, specialists and hospitalisation are lower for economic immigrants (24.94, 9.77 and 7.27% respectively) than for natives (30.68, 12.72 and 8.63%, respectively), for emergencies, 30.51% of economic immigrants reported having used the service, above the 25.32% for natives. When we disaggregate health care utilisation sample frequencies for each of the three years, we can observe that for general practitioner service, this proportion is always greater for natives than for economic immigrants, although this difference tends to be reduced over the decade. There is also a difference that favours natives’ access for specialist care which is reduced slightly during the period analysed. The proportion of hospitalisations was practically the same in 2006–07 and 2011–12, but inequality favouring the natives is observed in 2016–17. Only in case of emergencies do economic immigrants report a higher proportion of contacts throughout the decade, although this difference reduces slightly over the decade.

**Table 3:** Descriptive stats by type of immigration for each year and for the pooled sample

Type var.	Variable	Mean 2006–07	Mean 2011–12	Mean 2016–17	Mean 2006–2017
Primary care	Native Spanish	0.3282	0.2970	0.2881	0.3068
	Economic immig.	0.2427^a^	0.2444^a^	0.2601^b^	0.2494^a^
	Non-econ immig.	0.1961^a^	0.1844^a^	0.2102^a^	0.1971^a^
Specia-list care	Native Spanish	0.1338	0.1340	0.1122	0.1272
	Economic immig.	0.0988^a^	0.1050^a^	0.0908^a^	0.0977^a^
	Non-econ immig.	0.0854^a^	0.0813^a^	0.0912	0.0860^a^
Emergen-cies	Native Spanish	0.2558	0.2387	0.2632	0.2532
	Economic immig.	0.3225^a^	0.2844^a^	0.3037^a^	0.3051^a^
	Non-econ immig.	0.2004^a^	0.1693^a^	0.2159^b^	0.1966^a^
Hospitali-sation	Native Spanish	0.0918	0.0823	0.0827	0.0863
	Economic immig.	0.0852	0.0806	0.0538^a^	0.0727^a^
	Non-econ immig.	0.0790	0.0438^b^	0.0682	0.0663^b^

Comparison with Native Spanish, Chi-square test. ^a^ Significant at 1%, ^b^ Significant at 5%

Sample frequencies of utilisation of health care services can give an erroneous picture when we want to address horizontal equity in utilisation (or access). Indeed, this is what often happens. However, when we adjust by health care needs and other socioeconomic and demographic characteristics, the resulting adjusted inequality (or inequity) gives a completely different result (particularly given the younger and healthier profile of economic immigrants, as compared to Spaniards). The results of the four multilevel logistic regressions are presented in Table 4. The same set of covariates was kept in the four models, enhancing comparability.

**Table 4:** Hierarchical logistic regressions estimates (natives groups vs economic immigrant group)

Variable	Primary_care Mean (SE)	Specialist_care Mean (SE)	Emergencies Mean (SE)	Hospitalisation Mean (SE)
Female	0.1651^a^ (0.0201)	0.1504^a^ (0.0273)	0.1084^a^ (0.0204)	−0.2679^a^ (0.0339)
Age 16–25	Ref.	Ref.	Ref.	Ref.
Age 26–35	0.0846^c^ (0.0475)	0.2684^a^ (0.0687)	− 0.2292^a^ (0.0415)	0.1529 (0.0951)
Age 36–45	0.019 (0.0459)	0.2203^b^ (0.0662)	−0.7295^a^ (0.0413)	0.0883 (0.0908)
Age 46–55	0.0515 (0.0463)	0.2077^b^ (0.0665)	−1.0017^a^ (0.0429)	0.143 (0.09)
Age 56–65	0.2181^a^ (0.0477)	0.107 (0.0689)	−1.2301^a^ (0.0463)	0.1095 (0.0921)
Age 66–75	0.2891^a^ (0.0546)	−0.0319 (0.0772)	−1.2719^a^ (0.0554)	0.2837^b^ (0.1)
Age76–85	0.318^a^ (0.0581)	−0.2887^a^ (0.082)	−1.1518^a^ (0.0589)	0.3508^b^ (0.1031)
Age more than 85	0.0714 (0.0736)	−0.7069^a^ (0.1078)	−1.1339^a^ (0.0761)	0.3019^b^ (0.12)
Living in pair	0.0558^b^ (0.02)	0.1415^a^ (0.0269)	0.059^b^ (0.0205)	0.047 (0.0333)
Health bad-very bad	Ref.	Ref.	Ref.	Ref.
Health fair	−0.0479 (0.0327)	−0.3426^a^ (0.0366)	− 0.3827^a^ (0.0328)	−0.6589^a^ (0.0406)
Health good	−0.5323^a^ (0.0354)	−1.0711^a^ (0.043)	−0.9845^a^ (0.0364)	−1.628^a^ (0.0511)
Health very good	−0.9209^a^ (0.0453)	−1.5624^a^ (0.0615)	−1.3326^a^ (0.0453)	−2.2249^a^ (0.0847)
Hypertension	0.3355^a^ (0.0224)	0.0318 (0.0302)	0.1138^a^ (0.0249)	0.0442 (0.036)
Stroke	0.233^a^ (0.0563)	0.297^a^ (0.0656)	0.4627^a^ (0.0577)	0.7002^a^ (0.065)
Heart	0.1454^a^ (0.0336)	0.2202^a^ (0.0408)	0.326^a^ (0.0348)	0.4882^a^ (0.0433)
Diabetes	0.2588^a^ (0.0317)	0.1536^a^ (0.04)	0.1583^a^ (0.034)	0.178^a^ (0.0447)
Cholesterol	0.1186^a^ (0.023)	0.0086 (0.0301)	−0.0165 (0.025)	− 0.1247^b^ (0.0367)
Tumor	0.0202 (0.0445)	0.7148^a^ (0.0478)	0.1564^b^ (0.0464)	0.6695^a^ (0.0542)
Mental	0.1805^a^ (0.0269)	−0.0048 (0.0338)	0.0841^b^ (0.0281)	−0.1338^b^ (0.0413)
Rest chronics	0.0579^a^ (0.0048)	0.0592^a^ (0.0059)	0.0528^a^ (0.005)	−0.0012 (0.007)
Limitation 2 weeks	0.8318^a^ (0.026)	0.5888^a^ (0.0309)	0.5334^a^ (0.0262)	0.5308^a^ (0.0374)
Accidents 12 months	0.1126^b^ (0.0359)	0.1135^b^ (0.0443)	1.1771^a^ (0.0346)	0.3738^a^ (0.0506)
GHQ12	0.0113^b^ (0.0038)	0.0245^a^ (0.0046)	0.0278^a^ (0.0038)	0.0279^a^ (0.0053)
No studies	Ref.	Ref.	Ref.	Ref.
Primary studies	−0.0299 (0.0306)	0.0498 (0.0412)	−0.0381 (0.0333)	0.0782^c^ (0.0465)
Secondary studies	−0.0958^b^ (0.0327)	0.1788^a^ (0.0438)	−0.0277 (0.0352)	0.0962^c^ (0.0514)
University studies	−0.2451^a^ (0.0437)	0.2069^a^ (0.0582)	−0.1435^b^ (0.0455)	0.0739 (0.0735)
Low social class	Ref.	Ref.	Ref.	Ref.
Medium-low SC	−0.0444^c^ (0.0269)	0.0401 (0.0366)	0.0327 (0.0278)	0.0692 (0.0444)
Medium-high SC	−0.151^a^ (0.0315)	−0.005 (0.0427)	− 0.1423^a^ (0.0329)	−0.0381 (0.0531)
High social class	−0.399^a^ (0.0372)	−0.1368^b^ (0.0498)	− 0.1999^a^ (0.0378)	−0.0508 (0.0632)
Inactive	Ref.	Ref.	Ref.	Ref.
Employed	−0.1747^a^ (0.0293)	−0.1925^a^ (0.0387)	0.1237^a^ (0.0299)	−0.2461^a^ (0.0522)
Unemployed	−0.0467 (0.0385)	−0.1169^b^ (0.0513)	0.1629^a^ (0.0384)	−0.0193 (0.0668)
Retired	0.1121^b^ (0.0335)	0.1268^b^ (0.0433)	0.1305^a^ (0.0371)	0.0351 (0.0522)
Small municipality	0.0921^a^ (0.0195)	−0.0219 (0.0259)	0.0156 (0.0199)	−0.0225 (0.0321)
2006 ^c^ Native Spanish	Ref.	Ref.	Ref.	Ref.
2011 ^c^ Native Spanish	−0.0781^b^ (0.0247)	0.1275^a^ (0.0325)	0.0854^b^ (0.0258)	−0.0176 (0.0418)
2017 ^c^ Native Spanish.	−0.2095^a^ (0.0242)	−0.1569^a^ (0.0328)	0.2166^a^ (0.0249)	0.0228 (0.0398)
2006 ^c^ Economic immig.	−0.0922 (0.0622)	−0.1841^b^ (0.0867)	0.2177^a^ (0.0578)	0.086 (0.1087)
2011 ^c^ Economic immig.	0.081 (0.0686)	0.0149 (0.0949)	0.3088^a^ (0.0657)	0.1879 (0.1248)
2017 ^c^ Economic immig.	0.0378 (0.0606)	−0.2913^b^ (0.0902)	0.4108^a^ (0.0579)	−0.0752 (0.117)
2006^c^ Non-Econ immig.	−0.2691^b^ (0.1244)	−0.268 (0.1719)	− 0.2368^c^ (0.1279)	0.1691 (0.197)
2011^c^ Non-Econ immig.	−0.4382^b^ (0.1635)	−0.1443 (0.2183)	− 0.1329 (0.1659)	−0.5245 (0.352)
2017^c^ Non-Econ immig.	−0.2473^c^ (0.1436)	−0.2454 (0.2037)	0.1725 (0.1414)	0.0857 (0.2356)
Intercept	−0.9489^a^ (0.0757)	−1.9167^a^ (0.0947)	−0.1571^b^ (0.0726)	−1.7855^a^ (0.1143)
${\sigma}_c^2$	0.0237 (0.0085)	0.0165 (0.0065)	0.0186 (0.0069)	0.0092 (0.0045)
Log-likelihood (Wald Chi test *p*-value)	−36,721.823 (0.0000)	−23,221.755 (0.0000)	−35,196.145 (0.0000)	−15,934.602 (0.0000)
N	69,311	69,123	69,231	68,892

^a^ Significant at 1%, ^b^ Significant at 5%, ^c^ Significant at 10%

Need for health care is a key variable to explain the probability of using each of the four health care services. As expected, individuals reporting a worse state of health have a higher probability of using any of the four health services increases, with a clear gradient as the state of health worsens. The probability of accessing SNHS services is also greater in those individuals who suffer some of the chronic diseases. The only exceptions are having cholesterol (that is not associated with the probability of using specialist or emergencies care), hypertension (which is not related with specialist care or hospitalisations), tumor (which is not related with primary care) and mental health (that is not associated with the probability of visiting a specialist, although the variable GHQ12 is positively and significantly associated with all health care services).

As for non-need factors, regarding socioeconomic characteristics, in general they also have the expected signs and significance. It is well known that the effect of educational attainment is different for patient’s initiated demand services (GP or emergency services) and for specialist care: the higher the education level, the lower (higher) the probability to use the former (latter). Little or no statistically significant relationship is found between the education level or social class and hospitalisations. The probability to contact any health care service of the SNHS is negatively related with social class (not significant for hospitalisations). As compared with those inactive, being employed is negatively related with the probability to visit any health care service but positively related with the probability to use emergency services (the extent to which both are connected -i.e. the emergency services arise as a consequence of delayed or no treatment by primary or specialist care-, is not known). Something similar happens with the unemployed who visit the specialist less, but more the emergency services, than those inactive. Finally, the probability to contact any health care services is positively related with being retired (not significant for hospitalisations) which might be attributed to a lower opportunity cost of time of this population group.

**Fig. 1:**
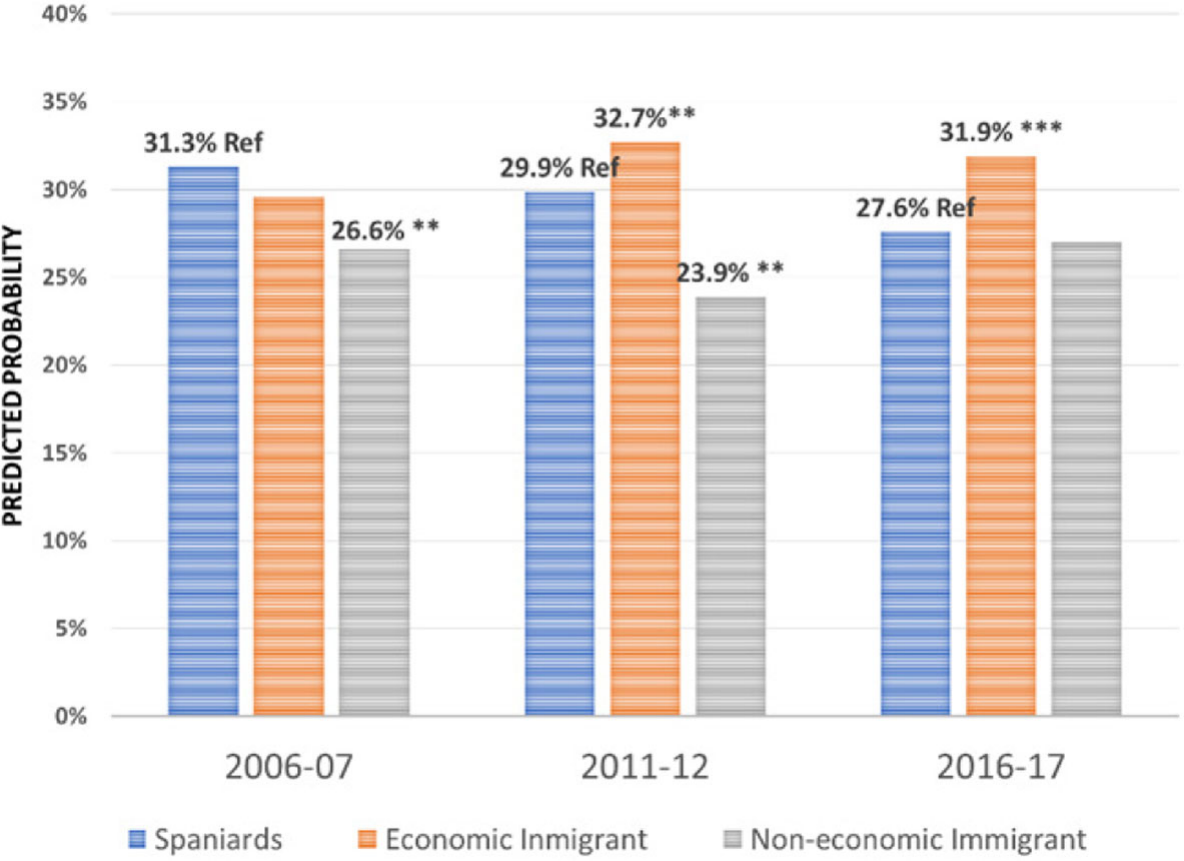
General Practitioner by economic condition

**Fig. 2:**
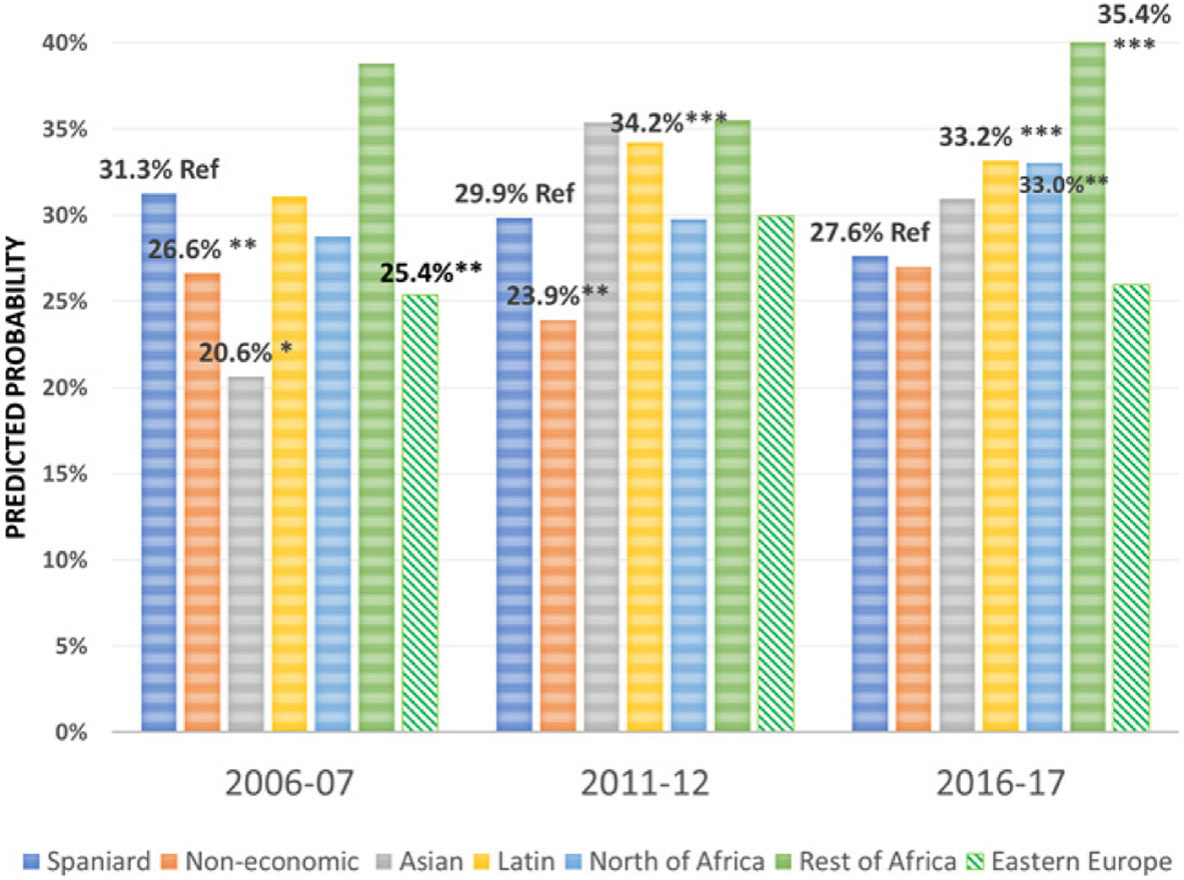
General Practitioner by continent of origin

**Fig. 3:**
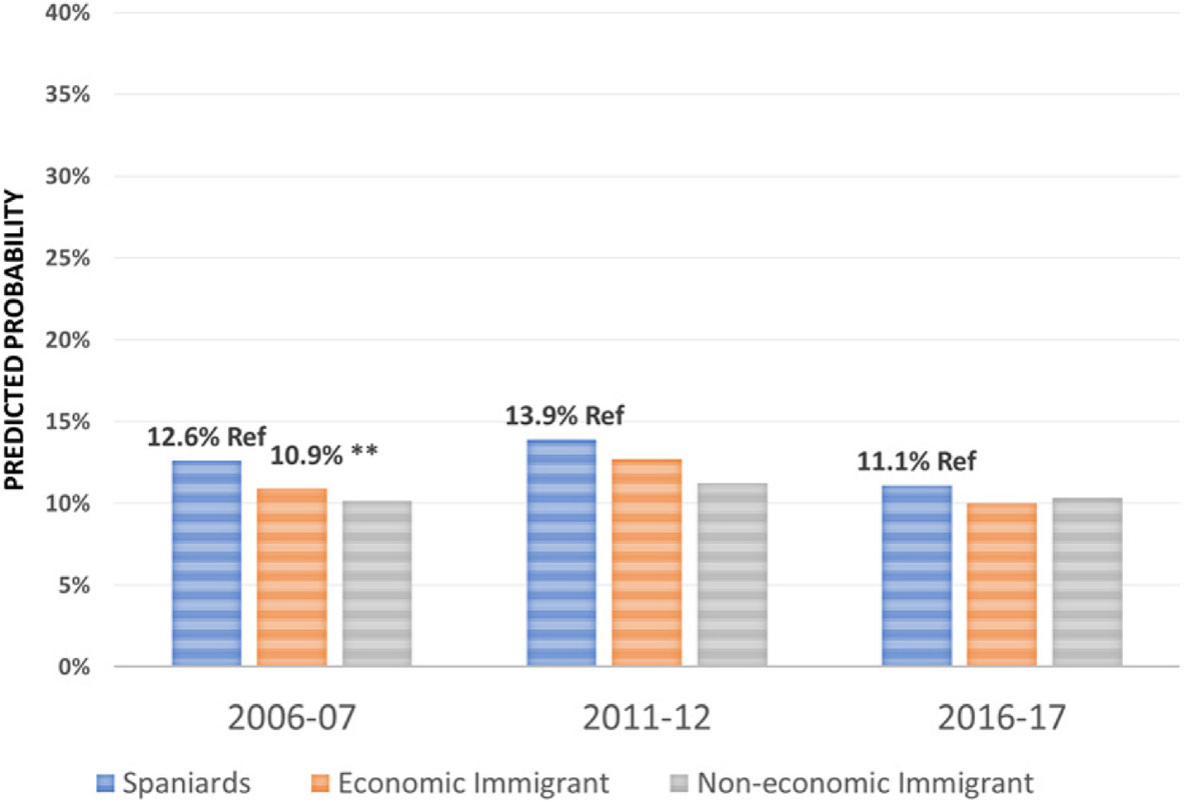
Specialist by economic condition

**Fig. 4:**
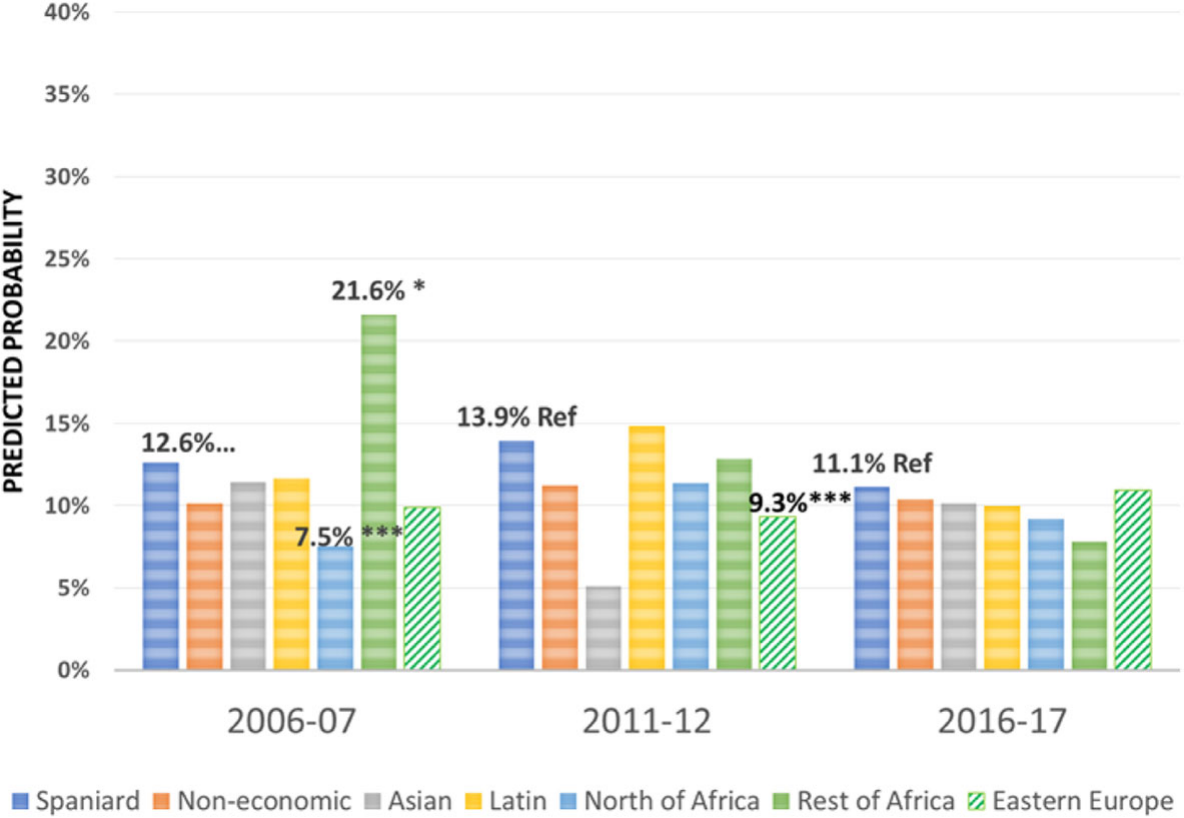
Specialist by continent of origin

**Fig. 5:**
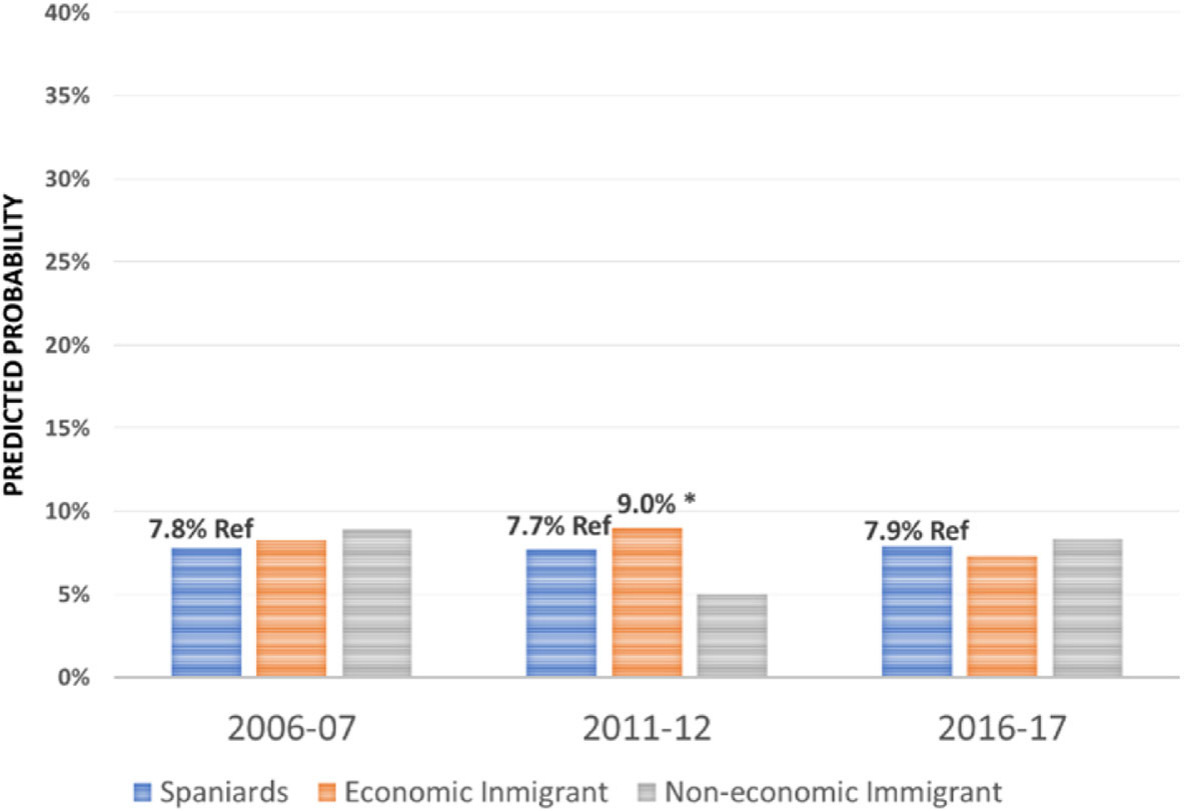
Hospitalisations by economic condition

**Fig. 6:**
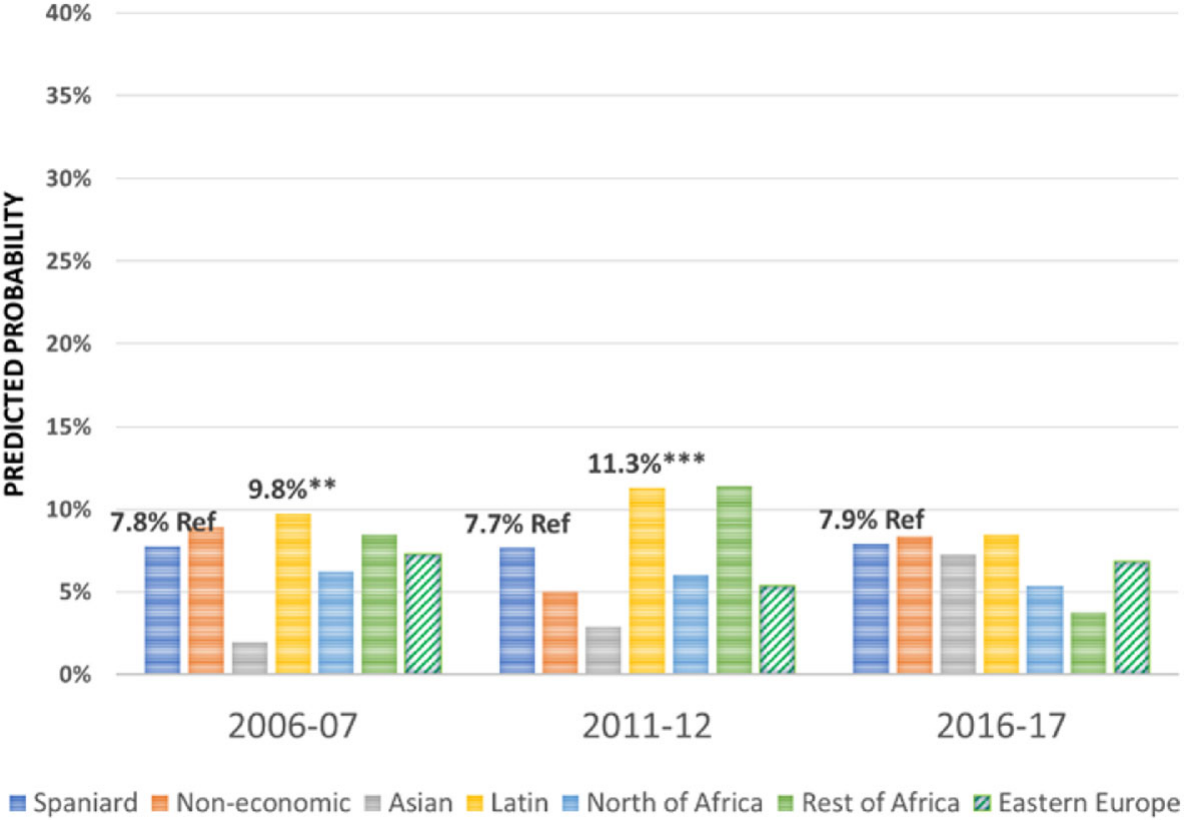
Hospitalisations by continent of origin

**Fig. 7:**
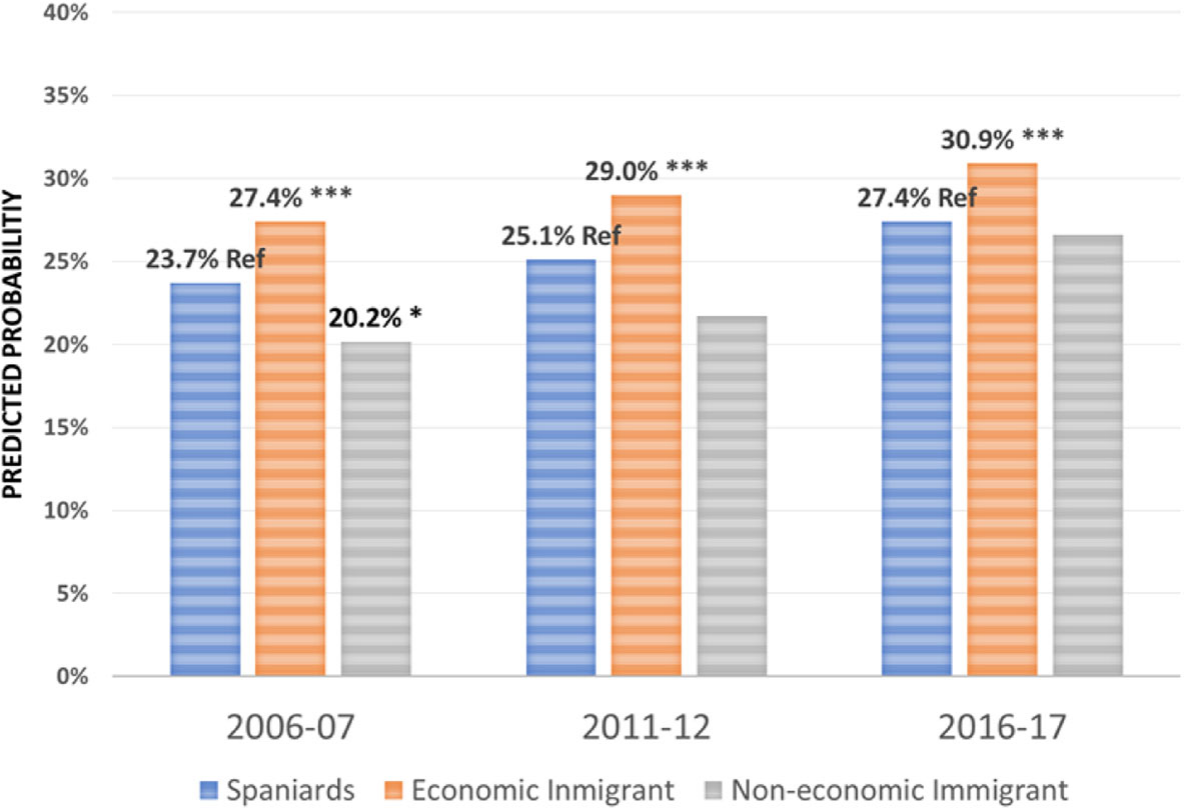
Emergencies by economic condition

**Fig. 8:**
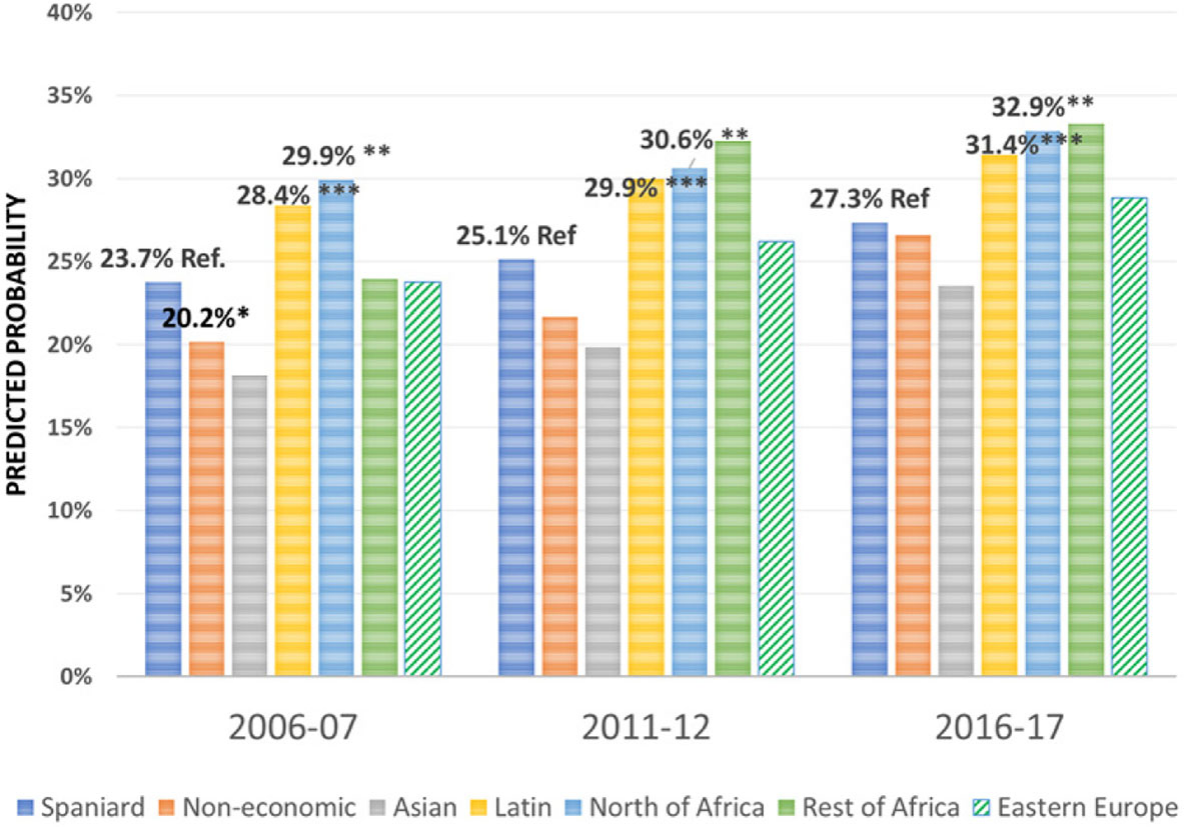
Emergencies by continent of origin

Regarding the effect of the immigrant condition on the probability of using the different health care services over the period 2006–2017 -the focus of this research-, the results are summarised in Figs. 1, 2, 3, 4, 5, 6, 7 and 8, where the predicted probabilities of each population group and year are presented. Each bar corresponds to a population group. Above each bar, the predicted probability is shown only if it is significantly different from the reference (which is the native Spanish population group). Statistical significance tests are shown in Additional file 1: Table S1 for the variant that considers just one economic immigrant group and in Additional file 1: Table S3 for the five-immigrant groups variant (the complete estimations for the five-economic immigrant groups variant can be seen in Additional file 1: Table S2). They are tests for horizontal inequity of each of the health care services in each year.

With respect to primary care services, in 2006–07, it can be seen that there are no global differences between predicted probabilities of use (i.e. no horizontal inequity in access), although an analysis by continent of origin shows some inequity that is detrimental to Eastern Europeans and Asians. However, a statistically significant difference that favours economic immigrants arises in 2011–12 that increases in 2017–18 (i.e. horizontal inequity favouring economic immigrants). By continent of origin, this difference is marked above all by Latin Americans since 2011–12, to which Africans join in 2017–18. There is no evidence of significant differences with respect to Eastern Europeans and Asians since 2011–2012. Regarding specialist services, 2006–07 shows that the predicted probability to visit a specialist doctor is lower for economic immigrants, particularly immigrants from North Africa (although immigrants from the rest of Africa show a higher predicted probability, *p* < 0.1) but this difference disappears with the economic crisis and after it (with the only exception of a lower probability to visit specialist doctor for Eastern Europeans in 2011–12). As for hospitalisations, in both 2006–07 and 2016–17, predicted probabilities of accessing hospital care are not different between natives and economic immigrants as a unique group (although, Latin Americans have a higher probability to use hospital services in 2006–07). However, in 2011–12, there is a difference in predicted probabilities that favours economic immigrants (again, concentrating it in Latin Americans). Predicted probabilities of using emergency services are significantly higher for economic immigrants over the three years. This difference is mainly driven by Latin Americans and those from North Africa (in fact, those from Asia and sub-Saharan Africa have similar access to that of the Spaniards).

Finally, the variance of the random effects at regional level is statistically significant, indicating differences in the probability of using each of the four health care services analysed related to being resident in one region or another.

## Discussion and conclusions

We find evidence of several horizontal inequities in access due to economic immigrant status once controlled for health care needs and socioeconomic and other demographic characteristics. Moreover, our results indicate that these inequities have changed throughout the 2006–2017 period. In addition, the effect of immigrant status is heterogeneous by continent of origin.

Patient-initiated health care services, such as general practitioner services, shows no evidence of horizontal inequity in access to primary care in at the beginning of the period in 2006–07 (results are in line with other previous studies [3, 6]). However, over time, inequity favouring economic immigrants arises, particularly in 2011, which is accentuated after the economic crisis in 2016–17. Thus, for patient-initiated health care services demand there has not been a relative worsening of immigrant access, rather the opposite. By continent, this difference is marked above all by immigrants from Latin America since 2011–12, who are then joined by those from Africa in 2016–17 (there are no differences in the access of Eastern Europeans and Asians with respect to Spaniards). This trend responds to an increase in the probability of using GP services by immigrants and a decrease in the probability of using these services by the autochthonous population (in line with the findings of [9] in their comparison of 2006–07 with 2011–12). There is also evidence of a reduction in GP visits during the economic crisis [17]. In their study, Urbanos-Garrido and Puig-Junoy analysed the interaction between social class and time trend during the economic crisis concluding that individuals who belong to the most disadvantaged social classes (including economic immigrants) have improved their access to public GP services during crisis times, as opposed to those belonging to more advantaged social classes [17]. In addition, employment conditions after the labour market reform in 2012 may explain the decrease in administrative visits to the GP of those with employment (with respect to those unemployed). During and after the start of the crisis, unemployment penalises immigrants more sharply than the native population, with the only exception being Asians (Blazquez and Herrarte [18],), who are precisely on of the group of economic immigrants whose propensity to use GP services is no different from that of Spaniards throughout the period analysed.

Something similar happens with emergency care, as there is inequity in access to emergency services that favours economic immigrants and that remains throughout the period. Again, this inequity benefits Latin Americans and those from North Africa (those from Eastern Europe, Asia and from Sub-Saharan Africa have similar access to that of Spanish natives). These results confirm the results of most previous evidence for 2006–07 [2–6], and for 2011–12 [9]. Some authors have related this evidence to knowledge about the functioning of the health care system by the immigrant population, but in two different manners. One explanation [3] is that immigrants know the way the health care system works well, so they anticipate the advantages of using emergency services to obtain fast and comprehensive diagnosis/treatment, avoiding in such a way the barriers they face to get access to specialist care (barriers also detected in such study) [3]. Another different explanation is that immigrants lack knowledge of the procedures to follow to access the rest of health care services lead to a higher propensity to use of emergency services [2]. The number of years that the immigrant has been living in Spain is relevant data that might also help to explain access patterns. This information, however, was not available for one the waves (2006–07), thus we could not include it in the model. A partial analysis for the waves 2011–12 and 2016–17 shows that the greater propensity to use emergency health services among the immigrant population is even greater for those who have lived in Spain for more than 5 years for 2016–17 (see Additional file 1: Table S4 and Figures S1 to S4). Thus, this result would give more support to the former argument.

Regarding doctor-initiated health care services and with respect to specialist care, 2006 starts with evidence of inequity that is detrimental to economic immigrants (particularly immigrants from North Africa), similar to results obtained previously [3–6]. This inequity has been attributed to unexplained differences associated with immigrants, rather than observed individual characteristics [7]. However, this inequity disappears with the economic crisis -as found by García-Subirats et al. [9]- (with the only exception of the Eastern Europeans) and after the crisis. One possible explanation might be that, as a consequence of the crisis and patients’ perception of longer waiting times for specialist care in the SNHS, the native population may have resorted relatively more to private specialist care, thus reducing the gap between the access of both population groups. This hypothesis deserves further research to be appropriately tested. With respect to hospitalizations, in 2006 and 2016, there is no evidence of inequity in access to hospital care for economic immigrants, with the only exception of Latin Americans who have a higher propensity to be hospitalised in 2006–07. Hernández-Quevedo and Jiménez-Rubio et al. [3] for 2006 also identified a larger probability of being hospitalised for Africans that we do not find (however, once again, they considered public and privately-funded health care services and this might explain this particular difference). Yet, in 2011–12, there is an inequity that favours economic immigrants. This relatively greater utilization of hospital services is concentrated amongst immigrants from Latin America. The higher birth rate in this population group may not fully explain this evidence either, since we have excluded births from hospital utilisation in our analysis (we have not been able to adjust for postpartum conditions that would be included in hospital utilisation data).

From a health policy viewpoint, the results obtained here may serve, firstly, to prevent alarm regarding a deterioration in the access to public health care services by economic immigrants (as a potentially vulnerable population) as a consequence of the economic crisis, and even after the implementation of the RD Law 16/2012. Furthermore, our results suggest that the inequity in access to specialist care that was found to be detrimental to economic immigrants in 2006, disappeared in 2011 and 2016. A thorough analysis of the heterogeneous implementation of the RD Law 16/2012 by regions in Spain might shed light on the possibility that a soft implementation of the RD by some regions prevented immigrants from new horizontal inequities in the access to the health care system. With respect to the horizontal inequity in emergency services that favours economic immigrants and remained throughout the decade, the fact that these services do not follow a similar pattern as that of specialist care may indicate that emergency services are still a popular way to access the system, particularly for those irregular immigrants who after the regulatory change of 2012 have just the right to use emergency health services.

Two additional points must be made. First, previous evidence presented above has considered total (public and privately- funded) health care services, thus, any comparison with our results must take this difference into account. If, as expected, utilisation of privately-funded health care services for economic immigrants was relatively lower than for native Spaniards, differences with respect to native Spaniards would have been larger than those shown in this research. Second, we must not forget that using a health care service (visiting a doctor, an emergency unit, etc.) does not guarantee an equally effective service, which is ultimately the relevant value of the utilisation of health services in the SNHS. The extent to which the quality of the health service could be explained at least partly by the immigrant condition is not known and would deserve more research.

There are some limitations in this study. First, given that this research is concerned with publicly-funded health care services, we have only been able to consider the last contact (visit, hospital admission, etc.) as a proxy for access. The degree to which our conclusions would remain if the frequency of contacts had been considered is not known. Second, for visits to the GP, specialist and emergency services, we have considered as publicly funded services those provided in public centres. However, it is possible that the service provided in a private centre is publicly funded (SNHS patients who are referred to agreed private centres for specialist visits, or mutualists who choose private providers), or even that the services provided in a public centre are privately paid (eg. out of pocket or by an insurance company), although this case is infrequent. We do not expect a relevant bias caused by the definition of the dependent variables, given that most health care services provided in agreed private centres are related to hospitalizations and diagnostic tests, and also given that the proportion of mutualists who choose private providers in the surveys -as compared with the whole samples- are 3.28% in 2006–07, 3.51% in 2011–12 and at 3.05% in 2016–17. Third, national health surveys do not distinguish the administrative situation of immigrants, that is, whether or not they are irregular immigrants. This information is crucial to address whether there are any differences in the access of both groups particularly after the implementation of the RD Law 16/2012. Fourth, there is an underrepresentation of economic immigrants in the different waves of the Spanish national health survey (particularly of Asians and Africans), so results obtained from these databases should be taken with caution. Fifth, our results regarding the variances of the random effects at regional level indicate that there is some variability across regions in the probability to use the four health care services analysed. We could not analyse whether there is a differential effect of the condition of economic immigrant by regions, due to the limitations with the sample size, but if a larger sample size was available, addressing this issue should be a priority, given the likely variability in regional policies regarding the provision of health care services to the immigrant population. Sixth, national health surveys do not include the institutionalized population (in hospitals, nursing homes, etc.), so results are subject to this restriction. Seventh, we have measured health care need with a set of self-reported measures of health state (self-assessed health status, whether the individual has any chronic condition, whether the individual has had any acute illness or accident and the GHQ-12 index of mental health). Despite the wide range of this set of health state measures, we may not have been able to pick up true clinical need, as judged by a clinician, let alone capacity to benefit from the health care system. Our results therefore must be taken with caution particularly if our self-reported measures significantly detach from an objective measure of health care need for the SNHS. Finally, we have used independent cross-sectional data, therefore, we have not been able to control for non-observed individual heterogeneity. The omission of individual characteristics that may affect the likelihood of using public health services might also have biased estimates of the immigration effect.

## Supplementary information

**Additional file 1: Table S1.** Hypothesis tests for the presence of inequities between native Spanish and the economic immigrant group. **Table S2.** Hierarchical logistic regression estimates (natives versus six economic immigrant groups). **Table S3.** Hypothesis tests for the presence of inequities between native Spanish and five economic immigrant groups. **Table S4.** Hierarchical logistic regression estimates (natives versus economic immigrant groups considering the years of living in Spain, equal/less or more than 5 years). **Figure S1.** Predicted probabilities of using General Practitioner services by immigrant condition. **Figure S2.** Predicted probabilities of using Specialist care services by immigrant condition. **Figure S3.** Predicted probabilities of using Hospitalisation services by immigrant condition. **Figure S4.** Predicted probabilities of using Emergency services by immigrant condition.

## Data Availability

All data are available in open in the following websites: http://www.mscbs.gob.es/estadEstudios/estadisticas/encuestaNacional/encuesta2006.htm http://www.mscbs.gob.es/estadEstudios/estadisticas/encuestaNacional/encuesta2011.htm http://www.mscbs.gob.es/estadEstudios/estadisticas/encuestaNacional/encuesta2017.htm
