# Supplementary material for: Trends in horizontal inequity in access to public health care services by immigrant condition in Spain (2006–2017)
Source: Int J Equity Health. 2019 Nov 29;18:185. doi: 10.1186/s12939-019-1092-1 (PMC6883664; doi:10.1186/s12939-019-1092-1)

**ADDITIONAL FILE**

Table S1. Hypothesis tests for the presence of inequities between native Spanish and the economic immigrant group

| Comparison  Native Spanish vs. | P-VALUE  2006-07 | P-VALUE  2011-12 | P-VALUE  2016-17 |
| --- | --- | --- | --- |
| Economic immig. | H0: 2006 * Economic immig. = 0 | H0: 2011 * Native Spanish =  2011 * Economic immig. | H0: 2016 * Native Spanish =  2016 * Economic immig. |
| Primary_care | 0.1378 | 0.0203** | <0.0001*** |
| Specialist_care | 0.0338** | 0.2342 | 0.1377 |
| Emergencies | 0.0002*** | 0.0007*** | 0.0008*** |
| Hospitalisation | 0.4287 | 0.0995* | 0.4015 |
| Non-Econ immig. | H0: 2006 * Non-Econ immig. = 0 | H0: 2011 * Native Spanish =  2011 * Non-Econ immig. | H0: 2016 * Native Spanish =  2016 * Non-Econ immig. |
| Primary_care | 0.0305** | 0.0276** | 0.7921 |
| Specialist_care | 0.1189 | 0.2129 | 0.6642 |
| Emergencies | 0.0642* | 0.1881 | 0.7551 |
| Hospitalisation | 0.3907 | 0.1499 | 0.7893 |

*** Significant at 1%, ** Significant at 5%, * Significant at 10%

Table S2. Hierarchical logistic regression estimates (natives versus six economic immigrant groups).

| VARIABLE | Primary_care  MEAN  (SE) | Specialist_care  MEAN  (SE) | Emergencies  MEAN  (SE) | Hospitalisation  MEAN  (SE) |
| --- | --- | --- | --- | --- |
| Female | 0.1661*** (0.0202) | 0.149*** (0.0273) | 0.1089*** (0.0205) | -0.2724*** (0.034) |
| Age 16-25 | Ref. | Ref. | Ref. | Ref. |
| Age 26-35 | 0.0854* (0.0476) | 0.2725*** (0.0688) | -0.2308*** (0.0415) | 0.165* (0.0952) |
| Age 36- 45 | 0.0172 (0.046) | 0.2229** (0.0663) | -0.7326*** (0.0414) | 0.098 (0.0909) |
| Age 46-55 | 0.0507 (0.0463) | 0.21** (0.0665) | -1.0047*** (0.0429) | 0.1515* (0.0901) |
| Age 56-65 | 0.2172*** (0.0478) | 0.1077 (0.0689) | -1.2324*** (0.0463) | 0.1156 (0.0921) |
| Age 66-75 | 0.2876*** (0.0546) | -0.0322 (0.0772) | -1.2732*** (0.0554) | 0.2875** (0.1001) |
| Age76-85 | 0.3167*** (0.0581) | -0.2905*** (0.082) | -1.1531*** (0.059) | 0.3525** (0.1032) |
| Age more than 85 | 0.0701 (0.0736) | -0.7094*** (0.1079) | -1.1346*** (0.0761) | 0.3036** (0.12) |
| Living in pair | 0.0574** (0.0201) | 0.1417*** (0.027) | 0.0598** (0.0205) | 0.0474 (0.0333) |
| Health bad-very bad | Ref. | Ref. | Ref. | Ref. |
| Health fair | -0.049 (0.0327) | -0.3437*** (0.0366) | -0.3834*** (0.0328) | -0.6612*** (0.0407) |
| Health good | -0.5344*** (0.0355) | -1.073*** (0.043) | -0.9865*** (0.0364) | -1.6303*** (0.0511) |
| Health very good | -0.9233*** (0.0453) | -1.5644*** (0.0615) | -1.3346*** (0.0453) | -2.228*** (0.0847) |
| Hypertension | 0.3357*** (0.0224) | 0.0314 (0.0302) | 0.114*** (0.0249) | 0.0446 (0.036) |
| Stroke | 0.2331*** (0.0563) | 0.2967*** (0.0656) | 0.4625*** (0.0577) | 0.701*** (0.065) |
| Heart | 0.1456*** (0.0336) | 0.2208*** (0.0408) | 0.3257*** (0.0348) | 0.4888*** (0.0433) |
| Diabetes | 0.2584*** (0.0317) | 0.1544*** (0.04) | 0.158*** (0.034) | 0.1789*** (0.0447) |
| Cholesterol | 0.119*** (0.023) | 0.0084 (0.0301) | -0.0164 (0.025) | -0.1251** (0.0367) |
| Tumor | 0.0201 (0.0445) | 0.7143*** (0.0479) | 0.1558** (0.0464) | 0.6676*** (0.0543) |
| Mental | 0.1814*** (0.0269) | -0.0047 (0.0338) | 0.0843** (0.0281) | -0.1335** (0.0413) |
| Rest chronics | 0.0578*** (0.0048) | 0.0591*** (0.0059) | 0.0526*** (0.005) | -0.0015 (0.007) |
| Limitation 2 weeks | 0.8305*** (0.026) | 0.5901*** (0.0309) | 0.5315*** (0.0262) | 0.5323*** (0.0374) |
| Accidents 12 months | 0.1122** (0.0359) | 0.1129** (0.0443) | 1.1773*** (0.0346) | 0.3735*** (0.0506) |
| GHQ12 | 0.0112** (0.0038) | 0.0241*** (0.0046) | 0.0276*** (0.0038) | 0.0274*** (0.0053) |
| No studies | Ref. | Ref. | Ref. | Ref. |
| Primary studies | -0.0295 (0.0306) | 0.0462 (0.0412) | -0.0363 (0.0333) | 0.0717 (0.0466) |
| Secondary studies | -0.0934** (0.0328) | 0.1732*** (0.044) | -0.0235 (0.0354) | 0.0853* (0.0517) |
| University studies | -0.2426*** (0.0438) | 0.2004** (0.0583) | -0.1394** (0.0456) | 0.0604 (0.0737) |
| Low social class | Ref. | Ref. | Ref. | Ref. |
| Medium-low SC | -0.042 (0.0269) | 0.0415 (0.0366) | 0.034 (0.0278) | 0.0708 (0.0445) |
| Medium-high SC | -0.1503*** (0.0316) | -0.0029 (0.0427) | -0.1411*** (0.0329) | -0.0339 (0.0531) |
| High social class | -0.3984*** (0.0372) | -0.1349** (0.0499) | -0.1993*** (0.0378) | -0.0467 (0.0633) |
| Inactive | Ref. | Ref. | Ref. | Ref. |
| Employed | -0.1753*** (0.0293) | -0.1948*** (0.0388) | 0.1243*** (0.0299) | -0.2505*** (0.0522) |
| Unemployed | -0.0469 (0.0385) | -0.1194** (0.0514) | 0.1623*** (0.0384) | -0.0196 (0.0669) |
| Retired | 0.1133** (0.0335) | 0.127** (0.0433) | 0.1313*** (0.0371) | 0.0348 (0.0522) |
| Small municipality | 0.0945*** (0.0195) | -0.0213 (0.0259) | 0.0157 (0.0199) | -0.022 (0.0321) |
| 2006 * Native Spanish | Ref. | Ref. | Ref. | Ref. |
| 2011 * Native Spanish | -0.0786** (0.0247) | 0.1279*** (0.0325) | 0.0849** (0.0258) | -0.0168 (0.0418) |
| 2017 * Native Spanish. | -0.2098*** (0.0242) | -0.1565*** (0.0328) | 0.2163*** (0.0249) | 0.0239 (0.0399) |
| 2006* Non-Econ immig. | -0.2691** (0.1244) | -0.2666 (0.1719) | -0.2374* (0.1279) | 0.1716 (0.197) |
| 2011* Non-Econ immig. | -0.4385** (0.1635) | -0.1429 (0.2183) | -0.1338 (0.1659) | -0.5219 (0.352) |
| 2017* Non-Econ immig. | -0.2475* (0.1436) | -0.2435 (0.2037) | 0.1715 (0.1414) | 0.0892 (0.2356) |
| 2006 * Eastern Europe | -0.3473** (0.1547) | -0.2994 (0.2191) | 0.001 (0.1349) | -0.0706 (0.2918) |
| 2011 * Eastern Europe | -0.0733 (0.149) | -0.3721 (0.233) | 0.1486 (0.1424) | -0.4354 (0.3646) |
| 2017 * Eastern Europe | -0.3098** (0.139) | -0.1762 (0.186) | 0.2996** (0.1246) | -0.1504 (0.2656) |
| 2006 *Asian | -0.6609* (0.387) | -0.1206 (0.4542) | -0.3827 (0.3646) | -1.5503 (1.044) |
| 2011 * Asian | 0.2223 (0.3175) | -1.0695 (0.7286) | -0.2605 (0.3573) | -1.1322 (1.0381) |
| 2017 * Asian | -0.0188 (0.2676) | -0.2688 (0.4051) | -0.0151 (0.2642) | -0.0831 (0.5205) |
| 2006 * Latin American | -0.0098 (0.0783) | -0.095 (0.1074) | 0.2741*** (0.0735) | 0.2827** (0.1314) |
| 2011 * Latin American | 0.1605* (0.0892) | 0.2106* (0.1164) | 0.3617*** (0.0855) | 0.4719** (0.1476) |
| 2017 * Latin American | 0.1042 (0.0817) | -0.2862** (0.121) | 0.4404*** (0.0782) | 0.1012 (0.1482) |
| 2006 * North Africa | -0.1431 (0.1424) | -0.6253** (0.2246) | 0.3606** (0.1293) | -0.262 (0.2546) |
| 2011 * North Africa | -0.085 (0.1712) | -0.1262 (0.2453) | 0.3997** (0.1566) | -0.3025 (0.3543) |
| 2017 * North Africa | 0.0972 (0.132) | -0.3859* (0.2124) | 0.5174*** (0.1254) | -0.4333 (0.3014) |
| 2006 * Rest Africa | 0.397 (0.3124) | 0.7251* (0.3812) | 0.0137 (0.3266) | 0.1017 (0.6134) |
| 2011 * Rest Africa | 0.2298 (0.3048) | 0.0236 (0.4736) | 0.487* (0.2875) | 0.4833 (0.5277) |
| 2017 * Rest Africa | 0.4728* (0.242) | -0.5834 (0.4734) | 0.5388** (0.2415) | -0.8388 (0.7325) |
| Intercept | -0.9513*** (0.0759) | -1.9108*** (0.0948) | -0.1582** (0.0725) | -1.7787*** (0.1144) |
| $\sigma_{c}^{2}$ | 0.0241 (0.0086) | 0.01672 (0.0066) | 0.0180 (0.0067) | 0.0094 (0.0046) |
| $Log-likelihood$  $(Wald Chi test p-value)$ | -36,710.311  (0.0000) | -23,211.496  (0.0000) | -35,187.14  (0.0000) | -15,922  (0.0000) |
| $N$ | 69,311 | 69,123 | 69,231 | 68,892 |

*** Significant at 1%, ** Significant at 5%, * Significant at 10%

Table S3. Hypothesis tests for the presence of inequities between native Spanish and five economic immigrant groups

| Comparison  Native Spanish vs. | P-VALUE  2006 | P-VALUE  2011 | P-VALUE  2016 |
| --- | --- | --- | --- |
| Eastern Europe | H0: 2006 * Eastern European = 0 | H0: 2011 * Native Spanish =  2011 * Eastern European | H0: 2016 * Native Spanish =  2016 * Eastern European |
| Primary_care | 0.0248** | 0.9718 | 0.4720 |
| Specialist_care | 0.1718 | 0.0317** | 0.9159 |
| Emergencies | 0.9938 | 0.6542 | 0.5035 |
| Hospitalisation | 0.8089 | 0.2508 | 0.5117 |
| Asian | H0: 2006 * Asian = 0 | H0: 2011 * Native Spanish =  2011 * Asian | H0: 2016 * Native Spanish =  2016 * Asian |
| Primary_care | 0.0877* | 0.3434 | 0.4757 |
| Specialist_care | 0.7906 | 0.1003 | 0.7818 |
| Emergencies | 0.2940 | 0.3337 | 0.3812 |
| Hospitalisation | 0.1376 | 0.2826 | 0.8371 |
| Latin American | H0: 2006 * Latin American = 0 | H0: 2011 * Native Spanish =  2011 * Latin American | H0: 2016 * Native Spanish =  2016 * Latin American |
| Primary_care | 0.9005 | 0.0074*** | 0.0001*** |
| Specialist_care | 0.3765 | 0.4768 | 0.2842 |
| Emergencies | 0.0002*** | 0.0012*** | 0.0041*** |
| Hospitalisation | 0.0314** | 0.0009*** | 0.6011 |
| North Africa | H0: 2006 * North Africa = 0 | H0: 2011 * Native Spanish =  2011 * North Africa | H0: 2016 * Native Spanish =  2016 * North Africa |
| Primary_care | 0.3148 | 0.9700 | 0.0203** |
| Specialist_care | 0.0054*** | 0.3002 | 0.2812 |
| Emergencies | 0.0053** | 0.0445** | 0.0165** |
| Hospitalisation | 0.3034 | 0.4200 | 0.1297 |
| Rest Africa | H0: 2006 * Rest Africa = 0 | H0: 2011 * Native Spanish =  2011 * Rest Africa | H0: 2016 * Native Spanish =  2016 * Rest Africa |
| Primary_care | 0.2039 | 0.3118 | 0.0048*** |
| Specialist_care | 0.0571* | 0.8256 | 0.3673 |
| Emergencies | 0.9667 | 0.1619 | 0.1818 |
| Hospitalisation | 0.8684 | 0.3433 | 0.2390 |
| Non-Econ immig. | H0: 2006 * Non-Econ immig. = 0 | H0: 2011 * Native Spanish =  2011 * Non-Econ immig. | H0: 2016 * Native Spanish =  2016 * Non-Econ immig. |
| Primary_care | 0.0306** | 0.0277** | 0.7928 |
| Specialist_care | 0.1209 | 0.2145 | 0.6696 |
| Emergencies | 0.0635* | 0.1874 | 0.7514 |
| Hospitalisation | 0.3838 | 0.1513 | 0.7812 |

*** Significant at 1%, ** Significant at 5%, * Significant at 10%

Table S4. Hierarchical logistic regression estimates (natives versus economic immigrant groups considering the years of living in Spain, equal/less or more than 5 years).

| VARIABLE | Primary_care  MEAN  (SE) | Specialist_care  MEAN  (SE) | Emergencies  MEAN  (SE) | Hospitalisation  MEAN  (SE) |
| --- | --- | --- | --- | --- |
| Female | 0.1762*** (0.0257) | 0.1699*** (0.0349) | 0.1233*** (0.0258) | -0.2605*** (0.0438) |
| Age 16-25 | Ref. | Ref. | Ref. | Ref. |
| Age 26-35 | 0.0914 (0.064) | 0.2244** (0.0935) | -0.2499*** (0.0558) | -0.07 (0.1311) |
| Age 36- 45 | -0.0162 (0.0611) | 0.1846** (0.0887) | -0.6603*** (0.0545) | -0.0435 (0.1213) |
| Age 46-55 | 0.0275 (0.0608) | 0.1946** (0.0882) | -0.9747*** (0.0559) | 0.0364 (0.1188) |
| Age 56-65 | 0.1793** (0.0615) | 0.1219 (0.0897) | -1.2104*** (0.0589) | 0.0851 (0.119) |
| Age 66-75 | 0.2391** (0.0709) | -0.0231 (0.1016) | -1.2429*** (0.0716) | 0.1673 (0.1313) |
| Age76-85 | 0.2676*** (0.0756) | -0.2654** (0.1078) | -1.1138*** (0.0762) | 0.3234** (0.1349) |
| Age more than 85 | 0.0287 (0.092) | -0.6088*** (0.1332) | -1.1943*** (0.0944) | 0.2019 (0.1528) |
| Living in pair | 0.0248 (0.0252) | 0.0859** (0.034) | 0.0186 (0.0255) | 0.0181 (0.0424) |
| Health bad-very bad | Ref. | Ref. | Ref. | Ref. |
| Health fair | -0.0845* (0.043) | -0.3102*** (0.0485) | -0.3582*** (0.0428) | -0.599*** (0.0537) |
| Health good | -0.5871*** (0.0463) | -1.1048*** (0.0564) | -0.96*** (0.0471) | -1.5586*** (0.0665) |
| Health very good | -0.9647*** (0.0584) | -1.5776*** (0.0788) | -1.3459*** (0.0581) | -2.196*** (0.1094) |
| Hypertension | 0.2873*** (0.0291) | 0.0304 (0.0394) | 0.1126*** (0.0317) | 0.0925** (0.0467) |
| Stroke | 0.139* (0.073) | 0.2096** (0.0875) | 0.3416*** (0.0751) | 0.5333*** (0.0867) |
| Heart | 0.0993** (0.0427) | 0.2163*** (0.0526) | 0.2989*** (0.0439) | 0.5676*** (0.0549) |
| Diabetes | 0.2571*** (0.0395) | 0.1176** (0.0507) | 0.1534*** (0.0421) | 0.1942** (0.056) |
| Cholesterol | 0.1257*** (0.0292) | 0.0022 (0.0387) | -0.0224 (0.0315) | -0.106** (0.0467) |
| Tumor | 0.0808 (0.0546) | 0.7251*** (0.0593) | 0.1793** (0.0564) | 0.6752*** (0.0671) |
| Mental | 0.1272*** (0.0358) | -0.0018 (0.0451) | 0.1102** (0.0367) | -0.1304** (0.0548) |
| Rest chronics | 0.0494*** (0.0062) | 0.0563*** (0.0076) | 0.0628*** (0.0064) | -0.0074 (0.0091) |
| Limitation 2 weeks | 0.831*** (0.0342) | 0.5557*** (0.0411) | 0.5698*** (0.0341) | 0.6053*** (0.049) |
| Accidents 12 months | 0.0792 (0.0571) | 0.1059 (0.0694) | 0.335*** (0.0564) | 0.3657*** (0.0795) |
| GHQ12 | 0.0118** (0.0048) | 0.0293*** (0.0058) | 0.0283*** (0.0048) | 0.029*** (0.0068) |
| No studies | Ref. | Ref. | Ref. | Ref. |
| Primary studies | -0.0073 (0.0423) | -0.0511 (0.0576) | -0.0531 (0.0453) | 0.0939 (0.0635) |
| Secondary studies | -0.0814* (0.0416) | 0.0972* (0.0558) | -0.0559 (0.0445) | 0.0472 (0.0647) |
| University studies | -0.1554** (0.0568) | 0.1976** (0.0755) | -0.1674** (0.0586) | 0.1153 (0.0957) |
| Low social class | Ref. | Ref. | Ref. | Ref. |
| Medium-low SC | -0.0444 (0.0343) | 0.0928* (0.0473) | 0.0295 (0.035) | 0.0626 (0.057) |
| Medium-high SC | -0.2347*** (0.0424) | 0.0197 (0.0578) | -0.1709*** (0.0433) | 0.0029 (0.0716) |
| High social class | -0.4505*** (0.0491) | -0.0815 (0.066) | -0.219*** (0.0491) | -0.1689** (0.0856) |
| Inactive | Ref. | Ref. | Ref. | Ref. |
| Employed | -0.1598*** (0.0402) | -0.1071** (0.0534) | 0.1876*** (0.0407) | -0.1351* (0.071) |
| Unemployed | -0.0838* (0.0484) | -0.0824 (0.0648) | 0.1946*** (0.048) | 0.0177 (0.0841) |
| Retired | 0.0827* (0.0439) | 0.0989* (0.0578) | 0.1307** (0.0485) | 0.0268 (0.0693) |
| Small municipality | 0.0586** (0.0252) | -0.0481 (0.0339) | -0.0063 (0.0254) | -0.0358 (0.0417) |
| 2011 * Native Spanish. | Ref. | Ref. | Ref. | Ref. |
| 2017 * Native Spanish. | -0.126*** (0.025) | -0.2840*** (0.0335) | 0.1236*** (0.0256) | 0.0275 (0.0418) |
| 2011 * Econ immig ≤ 5 | 0.0711 (0.1456) | 0.0835 (0.1948) | 0.2650** (0.1322) | 0.1824 (0.3030) |
| 2017 * Econ immig ≤ 5 | 0.0048 (0.1785) | -1.0921*** (0.3667) | 0.1681 (0.1645) | 0.1520 (0.3509) |
| 2011 * Econ immig > 5 | 0.0922 (0.0765) | -0.1814* (0.1070) | 0.1729** (0.0739) | 0.2286* (0.1362) |
| 2017 * Econ immig > 5 | 0.0549 (0.0643) | -0.3761*** (0.0938) | 0.3015*** (0.0614) | -0.0909 (0.1245) |
| 2011* Non-Econ immig. | -0.3778** (0.1628) | -0.2865 (0.2184) | -0.2371 (0.1653) | -0.5160 (0.3533) |
| 2017* Non-Econ immig. | -0.1785 (0.1429) | -0.3894* (0.2038) | 0.0780 (0.1410) | 0.1093 (0.2354) |
| Intercept | -0.8702*** (0.0938) | -1.9266*** (0.1585) | -0.0721 (0.0907) | -1.7873*** (0.1475) |
| $\sigma_{c}^{2}$ | 0.0256 (0.0094) | 0.0310 (0.0124) | 0.0218 (0.0083) | 0.0093 (0.0055) |
| $Log-likelihood$  $(Wald Chi test p-value)$ | -21,972.626  (0.0000) | -13,754.476  (0.0000) | -21,508.026  (0.0000) | -9,412.124  (0.0000) |
| $N$ | 41,849 | 41,826 | 41,833 | 41,653 |

*** Significant at 1%, ** Significant at 5%, * Significant at 10%

**FIGURES OF PREDICTED PROBABILITIES OF USING HEALTH CARE SERVICES BY IMMIGRANT CONDITION (economic immigrants with > < = 5 years living in Spain).**

Figure S1. General Practitioner Figure S2. Specialist


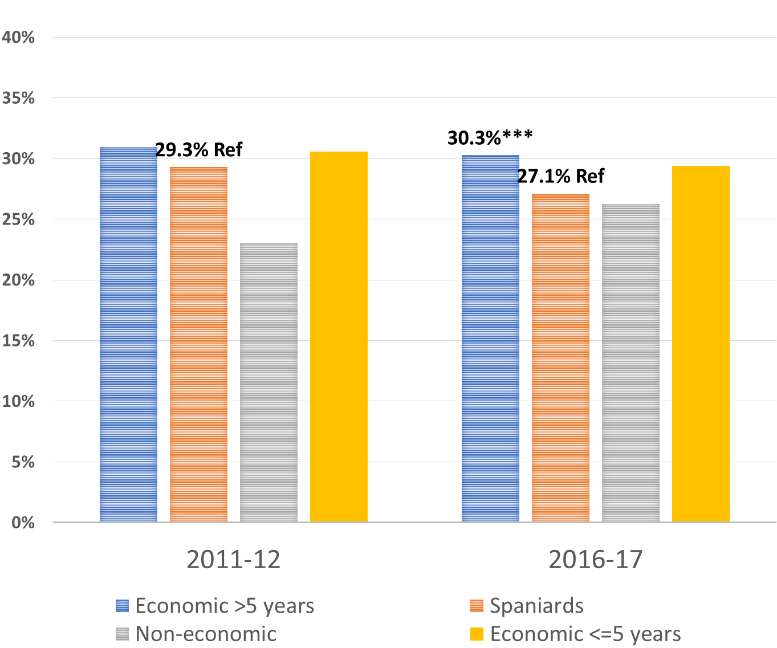

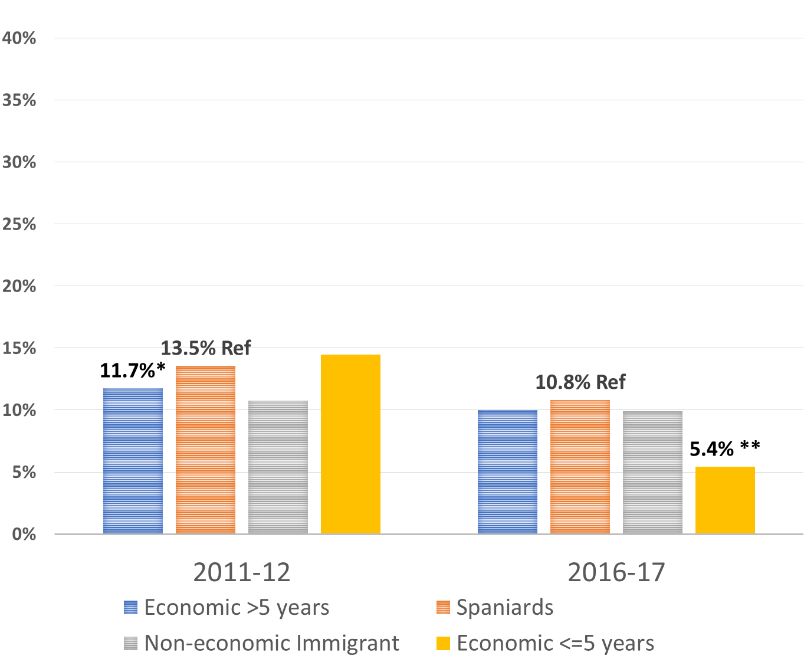


Figure S3. Hospitalisation Figure S4. Emergencies
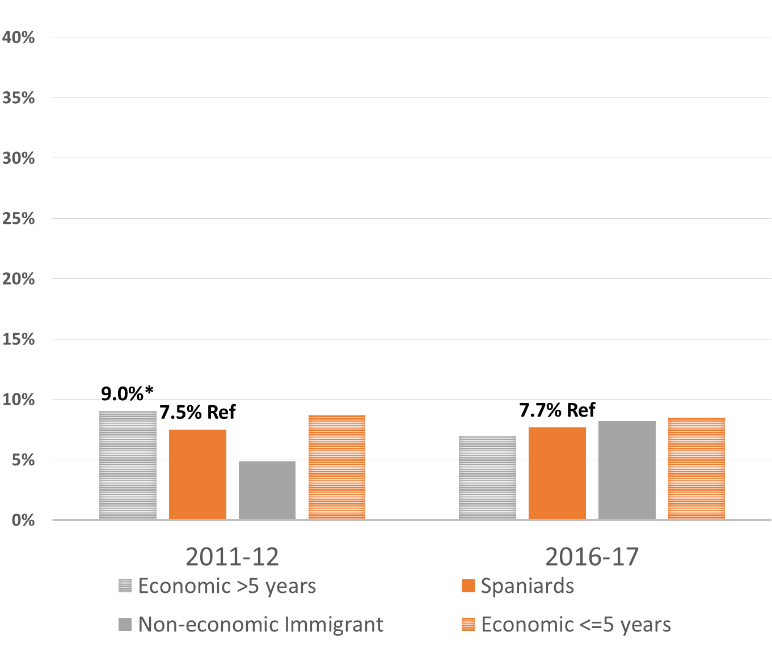

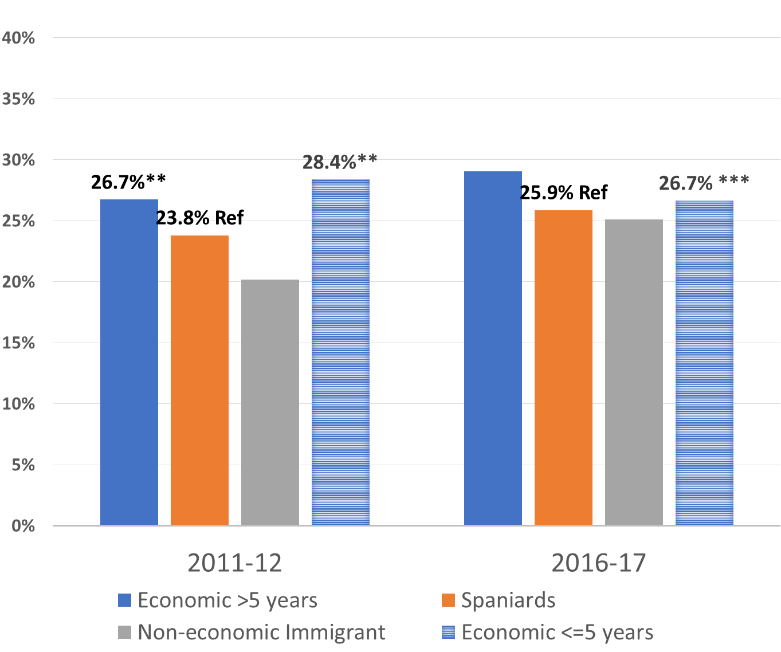

Supplement: Supplementary file 1 — Additional file 1: Table S1. Hypothesis tests for the presence of inequities between native Spanish and the economic immigrant group. Table S2. Hierarchical logistic regression estimates (natives versus six economic immigrant groups). Table S3. Hypothesis tests for the presence of inequities between native Spanish and five economic immigrant groups. Table S4. Hierarchical logistic regression estimates (natives versus economic immigrant groups considering the years of living in Spain, equal/less or more than 5 years). Figure S1. Predicted probabilities of using General Practitioner services by immigrant condition. Figure S2. Predicted probabilities of using Specialist care services by immigrant condition. Figure S3. Predicted probabilities of using Hospitalisation services by immigrant condition. Figure S4. Predicted probabilities of using Emergency services by immigrant condition. [file 12939_2019_1092_MOESM1_ESM.docx]
